# Synthesis Characterization and Antimicrobial Activity Studies of Some Transition Metal Complexes Derived from 3-Chloro-*N′*-[(1*E*)-(2-hydroxy phenyl)methylene]-6-methoxy-1-benzothiophene-2-carbohydrazide

**DOI:** 10.1155/2013/451629

**Published:** 2013-12-26

**Authors:** Vivekanand D. Biradar, B. H. M. Mruthyunjayaswamy

**Affiliations:** Department of Studies and Research in Chemistry, Gulbarga University, Gulbarga-585 106, Karnataka, India

## Abstract

A series of new coordination complexes of Cu(II), Co(II), Ni(II), Zn(II), Hg(II), Mn(II), and Fe(III) with the Schiff base 3-chloro-*N*′-[(1*E*)-(2-hydroxy phenyl)methylene]-6-methoxy-1-benzothiophene-2-carbohydrazide (HL) have been synthesized and characterized by elemental analysis, electrical conductivity measurements, IR spectra, ^1^H NMR, mass spectral data, electronic spectra, magnetic susceptibility, ESR spectra, TGA, and Powder XRD data. The Schiff base behaves as tridentate ONO donor ligand and forms the complexes of the type ML_2_ (metal-ligand) stoichiometry for Cu(II), Co(II), Ni(II), and Mn(II) complexes and ML stoichiometry for Zn(II), Hg(II), and Fe(III) complexes. All the complexes are colored and nonelectrolytes. It is found that Cu(II), Co(II), Ni(II), Mn(II) and Fe(III) complexes have exhibited octahedral geometry whereas Zn(II) and Hg(II) complexes exhibited tetrahedral geometry. The ligand and its metal complexes have been screened for their antibacterial activity against *E. coli* and *S. aureus* and antifungal activity against *A. niger* and *A. flavus.*

## 1. Introduction

Metal complexes with potentially tridentate and tetradentate ligands have evoked much interest in coordination chemistry [1]. Schiff base complexes of transition metals have played prominent role in the development of coordination chemistry [2]. Several Schiff base metal complexes have been studied because of their industrial and biological applications [3–5]. Schiff bases containing polyfunctional groups offer many practical advantages and unique structural environment for complexation [6]. Chen et al. [7] have reported Cu(II) complexes of thiophene-2,5-dicarboxylic acid with a view of constructing diverse low dimensional coordination polymers and the model coordination compounds. Literature survey reveals that many benzothiophene [8–10] derivatives are known to possess good biological activities like antimicrobial, anti-inflammatory, analgesic, diuretic, and antiviral activities.

In view of these findings and in continuation of our research work on coordination chemistry [11–16], we are reporting herewith the synthesis, characterization, and antimicrobial activity of Cu(II), Co(II), Ni(II), Zn(II), Hg(II), Mn(II), and Fe(III) complexes of 3-chloro-*N*′-[(1*E*)-(2-hydroxy phenyl)methylene]-6-methoxy-1-benzothiophene-2-carbohydrazide (HL) (Figure 9) in this communication (H refers to phenolate).

## 2. Experimental

### 2.1. Material and Method

All the chemicals are of reagent grade. Solvents were dried and distilled before use according to standard procedure [17]. The precursor 3-chloro-6-methoxy benzothiophene-2-carbohydrazide was prepared by literature method [18]. The absorption of metal and chloride containt of the complexes were carried out by the standard procedure. [19]. The metal chlorides used were in their hydrated form.

### 2.2. Synthesis of the Ligand HL

**Scheme 1:**
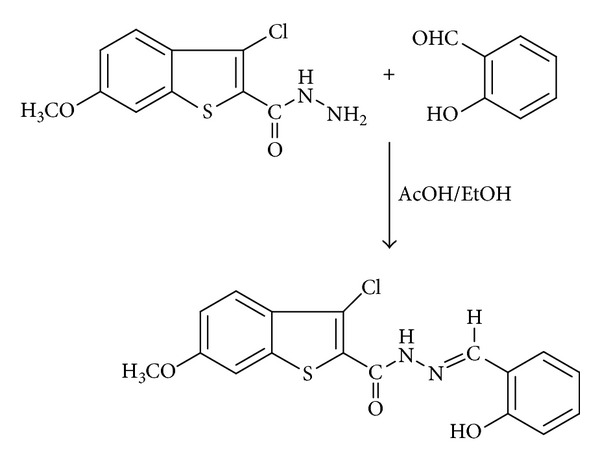
Synthesis of ligand HL.

An equimolar mixture of 3-chloro-6-methoxy-benzothiophene-2-carbohydrazide (0.001 mol) and salicylaldehyde (0.001 mol) in ethanol (30 mL) was refluxed in presence of catalytic amount of glacial acetic acid (1-2 drops) for about 6 h on water bath. The reaction mixture was cooled to room temperature, and the separated Schiff base (HL) (Figure 9) was collected by filtration, washed with ethanol, dried and recrystallized from absolute ethanol (Scheme 1).

### 2.3. Preparation of Cu(II), Co(II), Ni(II), Zn(II), Hg(II), Mn(II), and Fe(III) Complexes of Ligand HL

To the hot solution of 3-chloro-*N*′-[(I*E*)-(2-hydroxy phenyl)methylene]-6-methoxy-1-benzothiophene-2-carbohydrazide (HL) (Figure 10) (0.002 mol) in ethanol (30 mL) was added a hot ethanolic solution (10 mL) of respective metal chloride (0.002 mol); the reaction mixture was refluxed on a steam bath for 4 h, then sodium acetate (0.5 g) was added to it and refluxed for further 2 h. It was then poured into distilled water. The resulting solid complexes were collected by filtration, washed with sufficient quantity of distilled water, then with hot ethanol to apparent dryness, and dried in a vacuum over anhydrous calcium chloride in a desiccator (Table 1).

### 2.4. Physical Measurements

IR spectra of the synthesized ligand and its complexes were recorded as KBr pellets on Perkins-Elmer Spectrum One FT-IR spectrometer. ^1^H NMR spectra were recorded on a Bruker Avance 400 MHz spectrometer in DMSO using TMS as an internal standard. UV-visible spectra of the complexes were recorded on Elico-SL 164 spectrometer in the range 200–1000 nm in DMF solution (10^−3^ M). Mass spectrum of ligand was acquired on MASPEC system. Elemental analysis was obtained from HERAEUS C, H, N–O rapid analyzer. ESR measurement was carried out on a Bruker BioSpin Gmbh spectrometer working at microwave frequency of 9.903 GHz. The experiment was carried out by using diphenylpicrylhydrazyl (DPPH) as reference with field set at 3200 gauss. Magnetic susceptibility was determined by the Faraday method using a model 300 Lewis coil force magnetometer of tesla field strength at room temperature and the instrument was calibrated with [Hg Co(SNC)_4_] [20].

### 2.5. Antimicrobial Activity

The *in vitro* biological screening effects of the investigated compounds were tested against the bacterial species *E. coli *and* S. aureus,* fungal species *A. niger *and* A. flavus *by the cup plate method at 1 mg/mL concentration.

The bacterial and fungal cultures were inoculated in nutrient broth (inoculation medium) and incubated overnight at 37°C. Inoculated medium containing 24 h grown culture was added aseptically to the nutrient medium and mixed thoroughly to get a uniform distribution. This solution was poured (25 mL in each dish) into Petri dishes and then allowed to attain room temperature. Wells (6 mm in diameter) were punched carefully using a sterile cork borer and were filled with test solution 25 *μ*L. The plates were allowed to stand for an hour in order to facilitate the diffusion of the drug solutions, then the plates were incubated at 37°C for 24 h for bacteria and 48 h for fungi and the diameter of the zone of inhibition was measured [21]. The results were compared with those of standard drug streptomycin for bacterial and fluconazole for fungal activity of the same concentration as that of the test compounds under identical conditions.

### 2.6. Antioxidant Activity by DPPH Radical Scavenging Activity

The free radical-scavenging activity of the compounds was measured in terms of hydrogen donating or radical scavenging ability using the stable radical DPPH described by Blois method [22–24]. Briefly, stock solution of the sample (0.001 g/mL) was prepared by dissolving it in DMSO. 3 mL solutions of each with varying concentration (25–100 *μ*g) were prepared from the stock solution in methanol. Solution of DPPH (0.01 mM) in methanol was prepared and 1 mL of this solution was added to the above test solutions. The mixture was shaken vigorously and incubated for 30 min, and then the absorbance was measured at 517 nm. All the tests were run in triplicate and expressed as the mean ± standard deviation (S.D). Vit-C and Vit-E were used as standard or positive control, parallel to the test compound and in the absence of the test compound/standard used as the negative control. The capability to scavenge the DPPH radical was calculated using the fallowing equation:

(1) $$\begin{array}{c} \text{Percentage}\;\text{of}\;\text{scavenging}\;\text{activity}=\frac{A_{o}-A_{e}}{A_{o}}\times 100, \end{array}$$

where *A*_*o*_ corresponds to the absorbance of the negative control that is without sample; *A*_*e*_ is the absorbance of sample with complex or ligand.

## 3. Results and Discussion 

The physical and analytical data of the synthesized ligand HL and its Cu(II), Co(II), Ni(II), Zn(II), Hg(II), Mn(II), and Fe(III) complexes are given in Table 1. The molar conductance of the complexes was measured in DMF at 10^−3^ M concentration. Measured conductance values of these complexes are too low to account for their electrolytic behavior.

### 3.1. IR Spectrum of Ligand HL

In the IR spectrum of the ligand HL, a medium intensity band on 3351 cm^−1^ is assigned to *ν*_NH_ vibrations and a sharp band of strong intensity at 1651 cm^−1^ due to *ν*_C=O_ vibration, respectively [25]. A sharp intensity band observed at 1618 cm^−1^ is assigned to *ν*_C=N_ vibrations. Presence of intramolecular hydrogen bonded (O–H … N) vibration of phenolic –OH with nitrogen of the azomethine group of the Schiff base [26] is evidenced by the appearance of a broad band at 2901 cm^−1^. Vibrations because C–O was observed at 1235 cm^−1^. The band due to C–S–C of thiophene ring was found to appear at 1518 cm^−1^ (Table 2).

### 3.2. IR Spectra of Cu(II), Co(II), Ni(II), Zn(II), Hg(II), Mn(II), and Fe(III) Complexes of Ligand HL

Appearance of band in the region 3112–3334 cm^−1^ in the IR spectra of Cu(II), Co(II), Ni(II), Mn(II), and Fe(III) complexes of ligand HL is assigned to *ν*_NH_ of amide function [27]; further appearance of strong intensity bands in the region 1585–1635 cm^−1^ with a shift in the lower frequency side in these complexes by 16–66 cm^−1^ when compared to C=O of ligand which appear at 1651 cm^−1^ indicates the involvement of C=O oxygen atom in bonding with metal ions as such without undergoing enolization.

But in case of Zn(II) and Hg(II) complexes of ligand HL, disappearance of the band due to *ν*_NH_ of amide function [28] and carbonyl function which were found to appear at 3351 and 1651 cm^−1^, respectively, in case of ligand clearly indicates the enolization of carbonyl function and subsequently co-ordination of enolized carbonyl oxygen to Zn(II) and Hg(II) ion via deprotonation [27]. The fact of enolization of amide function was further conformed by the appearance of a new band (enhanced intensity) in the region 1601 cm^−1^ in the case of these Zn(II) and Hg(II) complexes. Medium intensity band which appeared at 1618 cm^−1^ in case of ligand HL was found to be shifted to lower frequency side by 58–83 cm^−1^ and appeared in the region 1535–1560 cm^−1^ in all these complexes of ligand HL suggesting the coordination of the nitrogen atom of the azomethine function of ligand with metal atoms. In the IR spectra of all these complexes, the absence of band due to intramolecular hydrogen bonded phenolic OH vibration which appeared at 2901 cm^−1^ in ligand clearly indicates the deprotonation of hydrogen bonded phenolic OH group during complexation with metal ions. The band due to phenolic C–O function observed at 1235 cm^−1^ in case of ligand HL has been found to be shifted to higher frequency side by 83–67 cm^−1^ in the complexes of the ligand HL under present study and appeared in the region 1302–1318 cm^−1^, confirms the coordination of phenolic oxygen with the metal ions *via* deprotonation.

The thiophene ring vibration of the ligand which appeared at 1518 cm^−1^ in case of ligand remained unaffected in all the complexes and appeared at about the same region 1501–1535 cm^−1^ which rules out any possibility of coordination by ring sulphur atom to metal ions.

Assignments in the far IR region are purely tentative because of various skeletal vibrations associated with metal ligand vibrations. The bands of weak intensity 564–505 cm^−1^ in case of all the complexes of ligand HL are assigned to *ν*_M−O_ vibrations. The absorption bands which appeared in the region 455–415 cm^−1^ in case of all the complexes are assigned to *ν*_M−N_ vibrations. In Zn(II), Hg(II), and Fe(III) complexes ligand HL, new bands observed in the region 315–312 cm^−1^ are assigned to *ν*_M−Cl_ vibrations. The broad band that appeared in the region 3417 cm^−1^ in case of Fe(III) complexes of ligand HL is assigned to *ν*_OH_ vibration of the coordinate or lattice water molecules. The IR spectral data of the ligand HL and its complexes are tabulated in Table 2.

### 3.3. ^1^H NMR Spectrum of the Ligand HL

**Figure 1:**
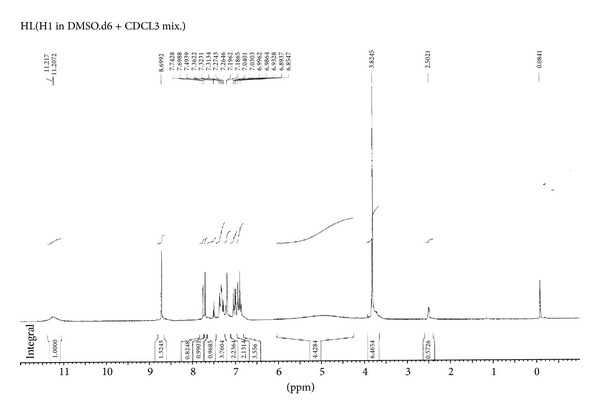
^1^H NMR spectrum of ligand HL.

The ^1^H NMR spectrum (in ppm *δ*) of the ligand HL (Figure 1) which displayed a fine broad singlet observed at 11.21 (s, 1H, OH) is assigned to intramolecular hydrogen bonded proton of OH group with azomethine nitrogen. Another broad singlet observed at 11.20 (s, 1H, CONH) is assigned to proton of CONH function. A sharp singlet at 8.69 (s, 1H, N=CH) is due to azomethine proton. The three protons of methoxy group have resonated as a sharp singlet at 3.82 (s, 3H, OCH_3_). Seven aromatic protons have resonated as multiplet in the region of 6.85–7.74 (m, 7H, ArH).

### 3.4. Mass Spectrum of the Ligand HL

**Figure 2:**
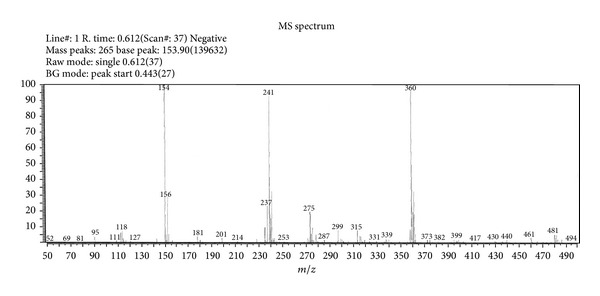
Mass spectrum of ligand HL.

**Scheme 2:**
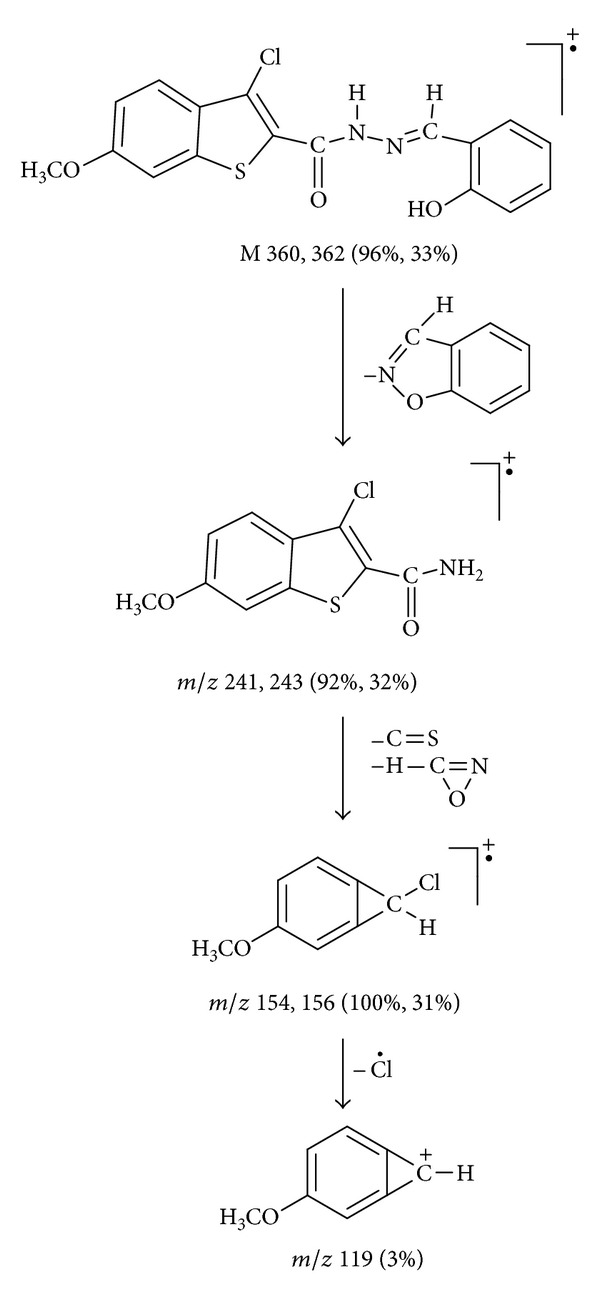
Mass fragmention pattern of ligand HL.

In the mass spectrum of the ligand HL (Figure 2), the molecular ion peak ${\text{M}}^{\underset{•}{+}}$ was observed at *m/z* 360, 362 (96%, 33%). The molecular ion underwent fragmentation to give fragment ions at *m/z* 241, 243 (92%, 32%) due to the expulsion of C_7_H_5_NO radical from the molecular ion. This fragment ion on further simultaneous loss of CS and HCNO species gave another fragment ion recorded at *m/z* 154, 156 (100%, 31%), which is also a base peak. This fragment ion further loses a chloride radical and gave a fragment ion recorded at *m/z* 119 (3%), (Scheme 2).

### 3.5. Electronic Spectra of Cu(II), Co(II), Ni(II), Mn(II), and Fe(III) Complexes of the Ligand HL

Electronic spectral data of the Cu(II), Co(II), Ni(II), Mn(II), and Fe(III) complexes of the ligand HL are given in Table 3. Electronic spectral studies of all these complexes were carried out in DMF at 10^−3^ M concentration.

#### 3.5.1. Cu(II) Complex

The electronic spectrum of Cu(II) complex derived from the ligand HL showed three bands at 13485, 18728, and 33870 cm^−1^. Rao et al. [29] has suggested octahedral geometry for Cu(II) complex due to Schiff base ligand. These complexes displayed a band in the region 13000–19000 cm^−1^ due to d-d transitions. Shashidhara et al. [30] have observed a broad band 15174 cm^−1^ to Cu(II) complex which is assigned to ^2^Eg-^2^T_2g_ and is a characteristic of distorted octahedral geometry. The observed broad band in the case of present Cu(II) complex of ligand HL can be assigned ^2^B_1g_ → ^2^E_g_, ^2^B_2g_, and ^2^A_tg_ transition suggesting distorted octahedral geometry of Cu(II) complexes.

#### 3.5.2. Co(II) Complex

Co(II) is d^7^ ion that exists both in octahedral and tetrahedral geometry. In octahedral Co(II) complexes three spin allowed transitions are expected corresponding to the transitions

1. ^4^T_1g_(F)→^4^T_2g_(F) (*ν*_1_) (~8000 cm^−1^)
2. ^4^T_1g_(F)→^4^A_2g_(F) (*ν*_2_) (~16000 cm^−1^)
3. ^4^T_1g_(F)→^4^T_2g_(P) (*ν*_3_) (~20000 cm^−1^).

Patel et al. [31] have reported three bands corresponding to *ν*_1_, *ν*_2_, and *ν*_3_ transition around 9000 cm^−1^, 14500 cm^−1^, and 20620 cm^−1^, respectively, for octahedral Co(II) complex. The Co(II) complex of the ligand HL under present study has showed three bands at 10772 cm^−1^, 15961 cm^−1^, and 19511 cm^−1^ due to ^4^T_1g_(F) → ^4^T_2g_(F) (*ν*_1_), ^4^T_1g_(F) → ^4^A_2g_(F) (*ν*_2_), and ^4^T_1g_(F) → ^4^T_2g_(P) (*ν*_3_) transition, respectively. These transitions suggest octahedral geometry for Co(II) complex.

#### 3.5.3. Ni(II) Complex

The ground state of Ni(II) in octahedral coordination is ^3^A_2g_(t_2g_^6^eg^2^). The Ni(II) complex shows three transitions in an octahedral field, namely,

 
^3^A_2g_(F) → ^3^T_2g_(F) (*ν*_1_) (7000–13000 cm^−1^)
 
^3^A_2g_(F) → ^3^T_1g_(F) (*ν*_2_) (11000–20000 cm^−1^)
 
^3^A_2g_(F) →^3^T_1g_(P) (*ν*_3_) (20000–27000 cm^−1^).

The bands in the region ~10000, ~12000, and ~25000 cm^−1^ ^3^A_2g_(F) →^3^T_1g_(P) (*ν*_3_), ^3^A_2g_(F) → ^3^T_1g_(F) (*ν*_2_), and ^3^A_2g_(F)→^3^T_2g_(F) (*ν*_1_) for the transitions mentioned above are characteristics of octahedral geometry.

The electronic spectrum of Ni(II) complex of the ligand HL under present investigation exhibited three bands in the region 10590 cm^−1^, 16598 cm^−1^, and 25617 cm^−1^ which are assigned to ^3^A_2g_(F)→^3^T_2g_(F) (*ν*_1_), ^3^A_2g_(F)→^3^T_1g_(F) (*ν*_2_), and ^3^A_2g_(F)→^3^T_1g_(P) (*ν*_3_) transition. All these observations favor the octahedral geometry for Ni(II) complex of the present study.

#### 3.5.4. Mn(II) Complex

The ground term of Mn(II) ion is in the sextet. The only sextet term of the d^5^ configuration in octahedral stereochemistry is the ^6^A_1g_. The transitions of the spectrum are assigned as from the ^6^A_1g_ ground term to the quartet excited term. Electronic absorption spectra of Mn(II) octahedral complex are expected to show four spin allowed transitions. The four narrow absorption bands approximately around 18000 cm^−1^, 24750 cm^−1^, 29500 cm^−1^, and 31900 cm^−1^ were assigned to ^6^A_1g_→^4^T_1g_(^4^G) (*ν*_1_), ^6^A_1g_  →^4^E_g_(^4^G) (*ν*_2_), ^6^A_1g_ →^4^E_g_(^4^D) (*ν*_3_), and ^6^A_1g_→^4^T_1g_(^4^P) (*ν*_4_) transitions, respectively, for octahedral Mn(II) complex [32]. The Mn(II) complex of the ligand HL under present study has exhibited four absorption bands at 17218, 23435, 25949, and 31434 cm^−1^, which corresponds to *ν*_1_, *ν*_2_, *ν*_3_, and *ν*_4_ transitions, respectively, suggesting octahedral geometry for the Mn(II) complex.

#### 3.5.5. Fe(III) complex

The electronic spectrum of Fe(III) complex displays three bands at 16429, 20524, and 25635 cm^−1^, which corresponds to *ν*_1_, *ν*_2_, and *ν*_3_. This may be assigned to ^6^A_1g_→^4^T_1g_,  ^6^A_1g_→^4^T_2g_, and ^6^A_1g_→^4^T_1g_, ^4^E_g_ transitions, respectively, typical of an octahedral geometry. These observed values for Fe(III) complex in its visible spectrum are in agreement with the literature values [33] and thereby proved to have octahedral geometry for the Fe(III) complex of the ligand HL.

### 3.6. Magnetic Susceptibility Data

Magnetic susceptibility measurements of the complexes were performed at room temperature. The magnetic moment for Cu(II) complex of the ligand HL is 1.91 BM. The reported value for the mononuclear Cu(II) having no major spin interaction is 1.75–2.20 BM [34, 35]. Thus the present Cu(II) complex is devoid of any spin interaction with distorted octahedral geometry. In octahedral Co(II) complex, the ground state is ^4^T_1g_ and a large orbital contribution to the singlet state lowers the magnetic moment values for the various Co(II) complexes which is in the range 4.70–5.20 BM. In the present investigation the observed magnetic moment value for Co(II) complex is 5.01 BM which indicates octahedral geometry for the Co(II) complex. For Ni(II) complex the observed magnetic moment value is 2.90 BM which is well within the expected range for Ni(II) complex with octahedral stereochemistry 2.83–4.00 BM [36, 37]. For Mn(II) and Fe(III) complexes the observed magnetic moment value is 5.68 BM and 5.91 BM, respectively, which are characteristic of octahedral geometry (Table 1).

### 3.7. ESR Spectral Studies of the Cu(II) Complex of the Ligand HL

The X-Band ESR spectrum of the powder Cu(II) complex was recorded at room temperature using DPPH as reference standard. One unpaired electron in Cu(II) complex with ^2^B_1g_ as ground state lies in d*x* ^2^-*y* ^2^orbital and follows the trend *g*_||_>*g*_⊥_>*g*_*e*_ (*g*_*e*_ = 2.0036 free ion value).

The observed *g*_||_ = 2.36, *g*_⊥_ = 2.07 values of the Cu(II) complex under the present study followed the same trend *g*_||_>*g*_⊥_>*g*_*e*_ which suggest the presence of unpaired electron in d*x* ^2^-*y* ^2^orbital giving octahedral geometry [35]. The observed *G* = 5.14 for the complexes under present study evidenced the monomeric nature of the complexes; this fact is further supported by the absence of a band corresponding to ΔMS = ± 2 transition in the observed ESR spectrum which is characteristic of monomeric complex (Table 3).

Consider

(2) $$\begin{array}{c} G=\frac{\left(G_{||}-2\right)}{\left(G_{⊥}-2\right)}\text{=}\;5.14. \end{array}$$

### 3.8. Thermogravimetric Analysis

**Figure 3:**
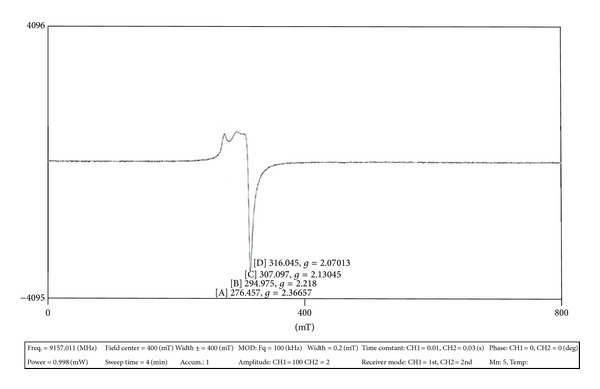
ESR spectrum of Cu(II) complex.

Thermogravimetry (TG) is a technique in which the change in mass of the sample is determined as a function of temperature and/or time. Among all the methods, TG is the most widely used one. From TG curve, information related to the thermal stabilities, composition of the initial sample, intermediate compounds that are formed, and the final residue could be obtained (Figure 3).

The TGA study on [Cu(HL)_2_] and [Co(HL)_2_] was carried out in the temperature range 38.69°C to 800°C.

#### 3.8.1. Cu(II) Complex

**Figure 4:**
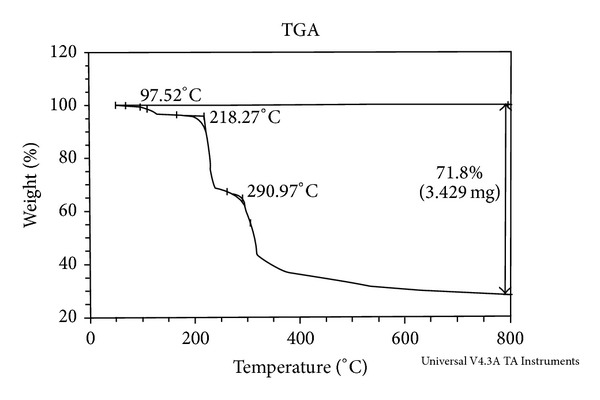
Thermogram of Cu(II) complex.

The decomposition studies of the compound Cu(II) complex [Cu(HL)_2_] have been carried out. In the thermogram of the [Cu(HL)_2_], (Figure 4) the first stage of the decomposition represents the weight loss of N_2_H_2_ at 97.52°C, with weight loss of 4.14%. The theoretical weight loss for this decomposition was 3.83% agreeing with observed value of 4.14%. The complex underwent further degradation and gave break at 218.27°C with a weight loss of 26.22%, which corresponds to the simultaneous decomposition of the C_9_H_6_OSCl and H_2_ species. This practical weight loss 26.22% is in accordance with theoretical weight loss of 25.47%. The third stage of decomposition occurs at 290.97°C, with weight loss of 25.53% which corresponds to the decomposition of C_9_H_6_OSCl species. This practical weight loss 25.53% is in accordance with theoretical weight loss of 25.80%. The fourth stage degradation at 328°C with weight loss of 16.73% corresponds to the decomposition of C_7_H_6_N_2_O species. This practical weight loss 16.73% is in accordance with theoretical weight loss 16.84%. Thereafter compound showed a gradual decomposition up to 800°C and onwards. The weight of the residue corresponds to copper oxide. The thermal decomposition of [Cu(HL)_2_] with probable assignments is given in Table 4.

#### 3.8.2. Co(II) Complex

**Figure 5:**
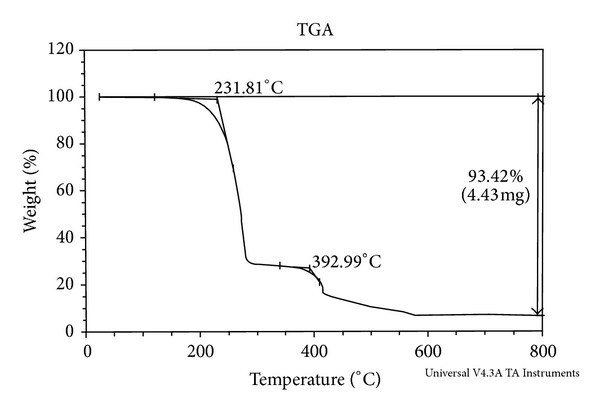
Thermogram of Co(II) complex.

In the thermogram of the [Co(HL)_2_], (Figure 5) the loss of C_26_H_18_N_2_O_4_S_2_Cl_2_ was observed at 231.81°C, with weight loss of 71.67%. This practical weight loss 71.67% is in accordance with the theoretical weight loss 71.50%. The resultant intermediate complex underwent further degradation and gave another break at 392.99°C with a weight loss of 12.53%, which corresponds to the simultaneous decomposition of the C_6_H_6_O and H_2_ species from the above intermediate complexes. The theoretical weight loss for this decomposition corresponds to 12.07% agreeing well with the observed value 12.53%. Thereafter compound showed a gradual decomposition up to 800°C and onwards. The weight residue corresponds to cobalt oxide (Co_3_O_4_). The thermal decomposition of [Co(HL)_2_] with probable assignments is given in Table 5.

### 3.9. Powder X-Ray Diffraction

#### 3.9.1. Cu(II) Complex

**Figure 6:**
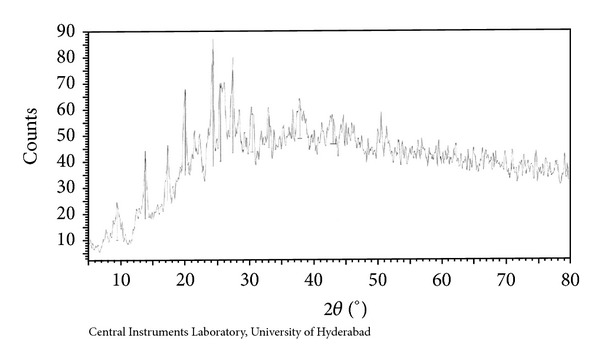
Powder X-ray diffraction of Cu(II) complex.

Powder X-ray diffraction pattern for Cu(II) complex of ligand HL has been depicted in Figure 6. The Copper complex of [Cu(HL)_2_] showed nine reflections in the range of 5–80° (2*θ*) arising from diffraction of X-ray by planes of complex. The interplanar spacing (*d*) has been calculated from the position of intense peak using Bragg's equation

(3) $$\begin{array}{c} \eta \lambda =2d\sin\theta , \end{array}$$

where *λ*  = wavelength of X-ray used (Cu K *α* = 1.54 Å).

The calculated spacing together with relative intensities with respect to most intense peak has been recorded in Table 6.

The 2*θ* value with maximum intensity of the peak for the compound was found to be 25.457 (2*θ*) which corresponds to *d* = 3.49612 Å. The 2*θ* values for the prominent peaks have been listed in Table 6. The entire important peaks have been indexed and unit cell calculations have been made for cubic symmetry of the complex. The observed values of interplanar distance have been compared with the calculated ones. It was observed that there is good agreement between the calculated and observed values. The experimental values of sin^2^ *θ* /common factor are recorded for each peak in Figure 6. The (*h* ^2^ + *k* ^2^ + *l* ^2^) values are 1, 2, 3, 4, 7, 10, 12, 15, and 20. The presence of forbidden no 7 and 15 indicates that the Cu(II) complex may belong to hexagonal or tetragonal system.

#### 3.9.2. Co(II) Complex

**Figure 7:**
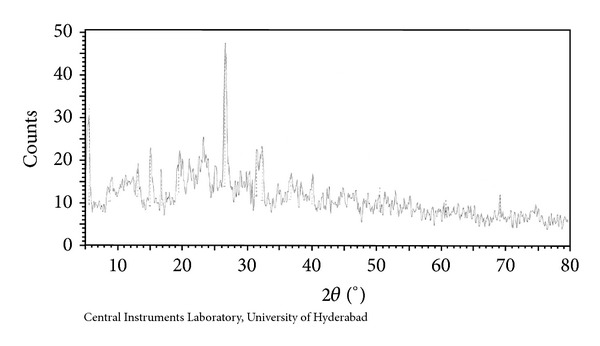
Powder X-ray diffraction of Co(II) complex.

Powder X-ray diffraction pattern for Co(II) complex of ligand HL has been depicted in Figure 7. The copper complex of [Co(HL)_2_] showed eleven reflections in the range of 5–80° (2*θ*) arising from diffraction of X-ray by planes of complex. The interplanar spacing (*d*) has been calculated from the position of intense peak using Bragg's equation

(4) $$\begin{array}{c} \eta \lambda =2d\sin\theta . \end{array}$$

The calculated spacing together with relative intensities with respect to most intense peak has been recorded in Table 7.

The 2*θ* value with maximum intensity of the peak for the compound was found to be 26.5511 (2*θ*) which corresponds to *d* = 3.35453 Å. The 2*θ* values for the prominent peaks have been listed in Table 7. The entire important peaks have been indexed and unit cell calculations have been made for cubic symmetry of the complex. The observed values of interplanar distance have been compared with the calculated ones. It was observed that there is good agreement between the calculated and observed values. The experimental values of sin^2^ *θ*/common factor are recorded for each peak in Figure 7. The (*h* ^2^ + *k* ^2^ + *l* ^2^) are 1, 2, 3, 5, 9, 12, 17, 20, 30, 43, and 54. The absence of forbidden no 7, 15, and 23 conforms the cubic symmetry. The experimental values are in good agreement with (*h* ^2^ + *k* ^2^ + *l* ^2^) values of primitive type cubic cell, with lattice parameter equal to *a* = *b* = *c* = 9.9518.

### 3.10. Antimicrobial Activity

Antimicrobial activity was carried out by the cup-plate method [33]. The ligand HL and its Cu(II), Co(II), Ni(II), Zn(II), Hg(II), Mn(II), and Fe(III) complexes have been tested for their antibacterial and antifungal activity at 1 mg/mL concentration. The results of the antimicrobial activity have been presented in Table 8. The results of the antibacterial activity testing showed that the complexes of Hg(II) and Mn(II) showed good activity, complexes of Cu(II), Co(II), Ni(II), and Fe(III) exhibited moderate activity, and ligand HLand complex of Zn(II) showed less activity against *E. coli* when compared with that of standard drug Streptomycin. The complexes of Hg(II) and Mn(II) showed good activity, and complexes of Cu(II), Co(II), Ni(II), and Fe(III) exhibited moderate activity against *S. aureus* when compared with that of standard drug Streptomycin.

The results of the antifungal activity testing of the ligand HL and its Cu(II), Co(II), Ni(II), Zn(II), Hg(II), Mn(II), and Fe(III) complexes showed that the complex of Hg(II) showed good activity and complexes of Co(II), Ni(II), and Mn(II) exhibited moderate activity against *A. niger* when compared with standard drug Fluconazole at the same concentration as that of the test compound. Ligand HL and complexes of Cu(II), Zn(II), and Fe(III) showed less activity. The complex of Hg(II) showed good activity, complexes of Ni(II), Zn(II), and Mn(II) exhibited moderate activity, and ligand HL and complexes of Cu(II), Co(II), and Fe(III) showed less activity against *A. flavus* when compared with standard drug Fluconazole.

### 3.11. Antioxidant Activity by DPPH Radical Scavenging Activity

**Figure 8:**
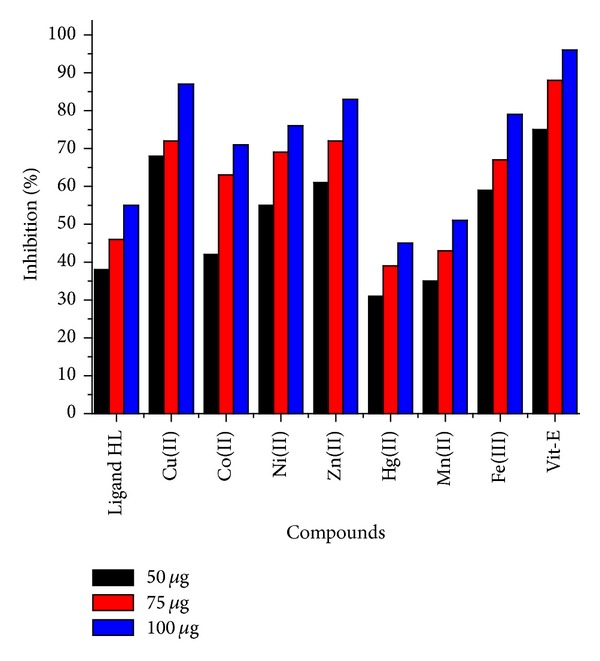
Antioxidant activities of ligand HL and its complexes.

**Figure 9:**
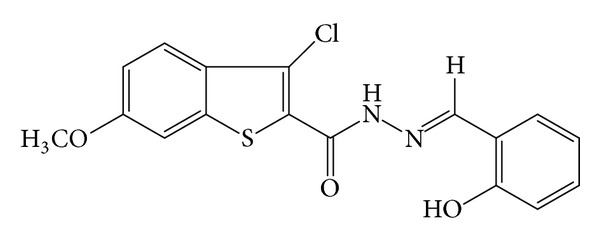
Schiff base HL.

**Figure 10:**
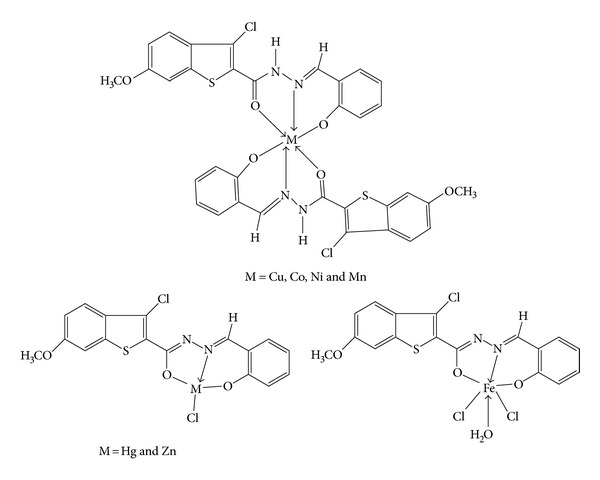
Proposed structure of the complexes of ligand HL.

DPPH scavenging activity of ligand and its complexes against vitamin-E as standard were analyzed at 50–100 *μ*g in DMF solution at *T* = 30 min. This investigation indicates that there is a great possibility of finding potent antioxidants. The Cu(II), Ni(II) Zn(II), and Fe(III) complexes have exhibited very good free radical scavenging activity, and Co(II) showed moderate activity. Ligand HL and its Hg(II) and Mn(II) complexes showed less activity compared with Vit-E. The bar graph representation of percentage of free radical scavenging activities is shown in Figure 8.

## 4. Conclusion

In the light of above discussion we have proposed that Cu(II), Co(II), Ni(II), Mn(II), and Fe(III) complexes have exhibited octahedral geometry whereas Zn(II) and Hg(II) complexes exhibited tetrahedral geometry. The ligand behaves as ONO tridentate chelating agent coordinating through the deprotonation of hydroxyl group, carbonyl group, and azomethine nitrogen. The elemental analysis, electrical conductivity measurements, IR spectra, ^1^H NMR, mass spectral data, electronic spectra, magnetic susceptibility, ESR spectra, and TGA revealed mononuclear nature of the complexes. The ligand HL was found to be less active against the bacteria as well as fungi whereas its complexes were found to be highly active against the bacteria as well as fungi and some of the complexes showed good antioxidant activity. On the basis of spectral evidence following structures have been assigned for synthesized complexes.

## Figures and Tables

**Table 1:** Physical, analytical, and magnetic susceptibility and molar conductance data of ligand HL and its complexes.

Compds	Molecular formula	Mol Wt.	M.P °C (yield in %)	Elemental analysis (%) Calcd (found)					Mag. moment *µ*_eff_ (BM)	Molar cond. (*ƛ*_M_) ohm^−1^ cm^−2^ mol^−1^	Colour
				M	C	H	N	Cl			
HL	C_17_H_13_N_2_O_3_SCl	360.81	266–268°C (65)	—	56.59 (56.41)	3.63 (3.53)	7.76 (7.85)	9.83 (9.60)	—	—	Colorless
Cu-complex	Cu(C_34_H_24_N_4_O_6_S_2_Cl_2_)	783.15	308–310°C (70)	8.11 (8.03)	52.14 (52.31)	3.09 (3.03)	7.15 (7.21)	9.05 (8.90)	1.91	12	Brown
Co-complex	Co(C_34_H_24_N_4_O_6_S_2_Cl_2_)	778.55	275–278°C (67)	7.32 (7.51)	52.45 (52.21)	3.11 (3.03)	7.20 (7.25)	9.11 (8.90)	5.01	14	Brown
Ni-complex	Ni(C_34_H_24_N_4_O_6_S_2_Cl_2_)	778.55	300 above (77)	7.54 (7.51)	52.47 (52.29)	3.11 (3.28)	7.20 (7.08)	9.11 (9.17)	2.90	33	Yellow
Zn-complex	Zn(C_17_H_11_N_2_O_3_SCl_2_)	459.67	222–225°C (62)	14.20 (14.10)	44.32 (44.10)	2.63 (2.41)	6.08 (5.91)	15.39 (15.29)	Diamagnetic	37	Yellow
Hg-complex	Hg(C_17_H_11_N_2_O_3_SCl_2_)	594.85	280–283°C (72)	33.66 (33.41)	34.27 (34.05)	2.03 (1.84)	4.70 (4.79)	11.90 (11.69)	Diamagnetic	11	Brown
Mn-complex	Mn(C_34_H_24_N_4_O_6_S_2_Cl_2_)	774.55	300 above (74)	7.09 (7.05)	52.72 (52.66)	3.12 (3.23)	7.23 (7.15)	9.15 (9.00)	5.68	16	Buff
Fe-complex	Fe(C_17_H_13_N_2_O_4_S_1_Cl_3_)	504.57	294–296°C (70)	11.07 (10.91)	40.47 (40.27)	2.80 (2.91)	5.55 (5.39)	21.08 (20.87)	5.91	10	Dark brown

**Table 2:** The IR (in cm^−1^) data of ligand HL and its complexes.

Compds	NH/NH	H/OH H_2_O	C=O	C=N	Phenolic C–O	C–S–C	C–O–C	>C=N–N=C<	M–Cl	M–N	M–O
Ligand HL	3351	2901	1651	1618	1235	1518	1268	—	—	—	—
Cu-complex	3267	—	1618	1535	1302	1501	1268	—	—	448	564
Co-complex	3112	—	1635	1551	1317	1501	1268	—	—	445	545
Ni-complex	3351	—	1618	1560	1318	1518	1269	—	—	455	510
Zn-complex	—	—	—	1535	1318	1535	1268	1601	312	418	551
Hg-complex	—	—	—	1551	1318	1535	1268	1601	315	424	519
Mn-complex	3334	—	1618	1151	1314	1518	1270	—	—	415	505
Fe-complex	3317	3417	1585	1551	1302	1502	1268	—	314	427	516

**Table 3:** Electronic and EPR spectral data of complexes of the ligand HL.

Compds	Electronic spectral data (in cm^−1^)				ESR data			
	*ν*_1_	*ν*_2_	*ν*_3_	*ν*_4_	*g*_⊥_	*g*_II_	*g*_av_	*G*
Cu-complex	13485	18728	—	33870	2.07	2.36	2.171	5.14
Co-complex	10772	15961	19511	—	—	—	—	—
Ni-complex	10590	16598	25617	—	—	—	—	—
Mn-complex	17218	23435	25949	31434	—	—	—	—
Fe-complex	16429	20524	25635	—	—	—	—	—

**Table 4:** Thermal decomposition of Cu(II) complex of ligand HL.

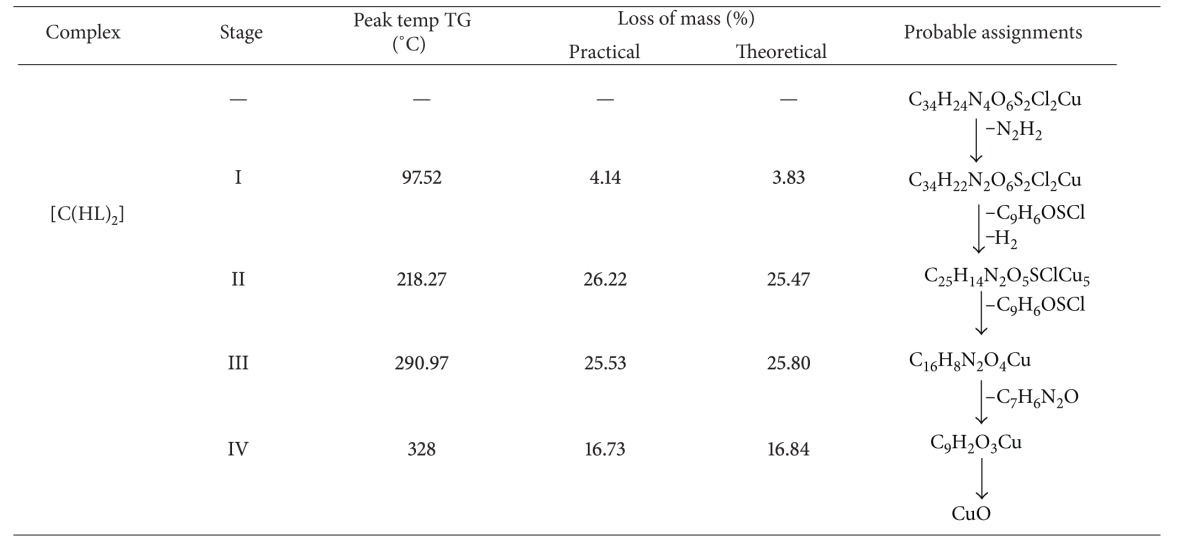

**Table 5:** Thermal decomposition of Co(II) complex of ligand HL.

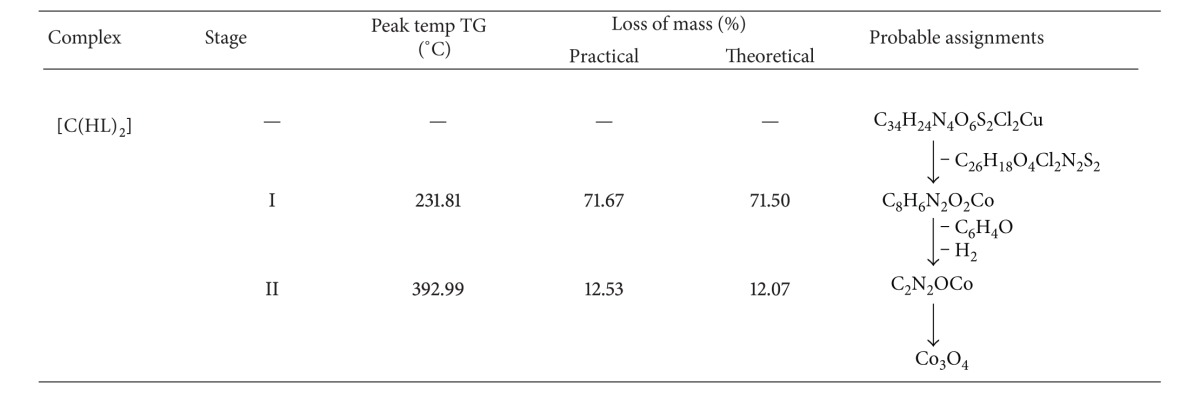

**Table 6:** Powder X-ray diffraction data of Cu(II) of the ligand HL.

Peak	2*θ*	*θ*	sin⁡⁡*θ*	sin⁡^2^⁡*θ*	*hk* *l*	*d*		*h* ^2^ + *k* ^2^ + *l* ^2^	*a* in Å
						Calc.	Obser.		
1	9.484	4.742	0.0826	0.00682	100	9.32567	9.32567	1	9.3277
2	13.899	6.9495	0.12099	0.01464	110	6.36664	6.36664	2	9.3277
3	17.399	8.6995	0.15125	0.02288	111	5.09289	5.09289	3	9.3277
4	20.154	10.077	0.17497	0.03061	200	4.40247	4.40247	4	9.3277
5	25.457	12.7285	0.22033	0.04855	—	3.49612	3.49612	7	9.3277
6	30.446	15.233	0.26257	0.06894	310	2.93369	2.93369	10	9.3277
7	33.056	16.528	0.28448	0.08093	222	2.70775	2.70775	12	9.3277
8	37.844	18.922	0.32428	0.10516	—	2.37542	2.37542	15	9.3277
9	42.952	21.476	0.36611	0.13404	420	2.10401	2.10401	20	9.3277

**Table 7:** Powder X-ray diffraction data of Co(II) complex of the ligand HL.

Peak	2*θ*	*θ*	sin⁡⁡*θ*	sin⁡^2^⁡*θ*	*hk* *l*	*d*		*h* ^2^ + *k* ^2^ + *l* ^2^	*a* in Å
						Calc.	Obser.		
1	8.8805	4.44025	0.07742	0.00599	100	9.94963	9.94939	1	9.9518
2	13.0916	6.5458	0.1139	0.01299	110	6.75761	6.75698	2	9.9518
3	15.0632	7.5316	0.1310	0.01718	111	5.87701	5.87671	3	9.9518
4	19.5309	9.7654	0.1696	0.02877	210	4.54133	4.54133	5	9.9518
5	26.5511	13.2755	0.2296	0.05273	300	3.35453	3.35438	9	9.9518
6	30.7545	15.3772	0.2651	0.07032	222	2.90493	2.90481	12	9.9518
7	36.8253	18.4126	0.3158	0.09977	410	2.43874	2.43869	17	9.9518
8	40.0795	20.0397	0.3426	0.11742	420	2.24794	2.24787	20	9.9518
9	50.5420	25.2710	0.4269	0.18224	521	1.8044	1.80436	30	9.9518
10	60.7614	30.3807	0.5057	0.25577	533	1.52311	1.52306	43	9.9518
11	69.1800	34.5900	0.5677	0.32228	721	1.35688	1.35684	54	9.9518

**Table 8:** Antimicrobial activity screening data of the ligand HL and its complexes.

Ligand/complexes	Antimicrobial activity (zone of inhibition in mm)*			
	Antibacterial activity		Antifungal activity	
	*E. coli *	*S. aureus *	*A. niger *	*A. flavus *
HL	12	13	11	10
Cu-complex	16	16	11	13
Co-complex	17	16	14	12
Ni-complex	17	17	15	14
Zn-complex	14	15	12	14
Hg-complex	21	20	18	17
Mn-complex	18	18	14	15
Fe-complex	17	16	12	11
Streptomycin	22	21	—	—
Fluconazole	—	—	19	20
DMF (control)	0	0	0	0

*Bore size-6 mm.
